# Supplementary material for: The genome sequence of the Large Birch Bell moth, Large Birch Roller, Epinotia brunnichana (Linnaeus, 1767)
Source: Wellcome Open Res. 2025 Feb 24;10:101. [Version 1] doi: 10.12688/wellcomeopenres.23749.1 (PMC11959263; doi:10.12688/wellcomeopenres.23749.1)
Supplement: Peer review history

# Reviewer report: Reviewer response for version 1

**DOI:** 10.21956/wellcomeopenres.26197.r119500

**Published:** 2025-03-31

**Authors:** Marko Mutanen

**Affiliations:** 1 University of Oulu, Oulu, Finland

This paper provides a genome sequence for a very common and widely distributed leaf roller species which belong to a genus with many species, including seemingly close relatives ( *E. solandriana, E. capreana, E. sordidana *etc.). There is no question about the identity of the species (as the photograph indicates), and this is also confirmed by DNA barcoding. The laboratory work and bioinformatics follow the DTOL standards, and as far as I can see, the genome is of high quality as indicated e.g. by the high BUSCO recovery rate. The main problem in the paper is that it does not introduce the species itself nearly to the degree I have seen done before with similar reports. There is a plenty of information available for the distribution, life history, phenology etc. for this species.

Are sufficient details of methods and materials provided to allow replication by others?

Yes

Is the rationale for creating the dataset(s) clearly described?

Partly

Are the datasets clearly presented in a useable and accessible format?

Yes

Are the protocols appropriate and is the work technically sound?

Yes

Reviewer Expertise:

Lepidoptera and Symphyta diversity, phylogenetics, molecular taxonomy, DNA barcoding, genomics

I confirm that I have read this submission and believe that I have an appropriate level of expertise to confirm that it is of an acceptable scientific standard, however I have significant reservations, as outlined above.

# Reviewer report: Reviewer response for version 1

**DOI:** 10.21956/wellcomeopenres.26197.r120149

**Published:** 2025-03-11

**Authors:** Kay Lucek

**Affiliations:** 1 University of Neuchâtel, Neuchâtel, Switzerland

The authors present the chromosome level genome assembly of a female specimen of the Large Birch Bell or Large Birch Roller *Epinotia brunnichana*. The assembly consists of 29 chromosomes and is not annotated for genes. The assembly is highly complete as revealed by the high BUSCO score but not fully phased. Sequencing and genome assembly follow the current state of the art and use established methods. The assembly has been generated using the established pipelines of the Darwin Tree of Life Consortium.

 Overall, the presented assembly will be of great value to study genome evolution given the relatively large genome size of 943 Mbps. Surprisingly there is no information about the biology of the species or its distribution in the UK and beyond. If such information is missing, it would be nice to have it highlighted.

 According to Wikipedia, the species is found in most western, central and northern Europe, the Near East up to the eastern Palearctic realm, being recorded from Russia, Kazakhstan, China, and Japan. Adults fly from July to October depending on the location. The larvae roll the leaves of Betula, Corylus and Salix and feed inside.

 On a technical side, it is not fully clear whether poly-G tails were removed or accounted for with the RNAseq data.

Are sufficient details of methods and materials provided to allow replication by others?

Yes

Is the rationale for creating the dataset(s) clearly described?

Yes

Are the datasets clearly presented in a useable and accessible format?

Yes

Are the protocols appropriate and is the work technically sound?

Yes

Reviewer Expertise:

Speciation genomics

I confirm that I have read this submission and believe that I have an appropriate level of expertise to confirm that it is of an acceptable scientific standard.

# Reviewer report: Reviewer response for version 1

**DOI:** 10.21956/wellcomeopenres.26197.r120147

**Published:** 2025-03-11

**Authors:** Stephan Koblmüller

**Affiliations:** 1 University of Graz, Graz, Austria

In this manuscript, Boyes et al. present the genome assembly of the Large Birch Bell moth, *Epinotia brunnichana*, a totricid moth widely distributed in Eurasia. Typically, some background information is given on the sequenced species, as well as a rationale for sequencing it. The rationale is given here (part of the Darwin tree of Life project), but some general background information on the species is entirely lacking. I suggest adding a few words/sentences on the species itself.

 The data are clear; the methods are well described. I have no objections to any of the data presented or the results. As far as I can tell, the datasets are deposited in / accessible from publicly accessible repositories.

Are sufficient details of methods and materials provided to allow replication by others?

Yes

Is the rationale for creating the dataset(s) clearly described?

Yes

Are the datasets clearly presented in a useable and accessible format?

Yes

Are the protocols appropriate and is the work technically sound?

Yes

Reviewer Expertise:

phylogenetics, population genetics, phylogeography, evolutionary biology

I confirm that I have read this submission and believe that I have an appropriate level of expertise to confirm that it is of an acceptable scientific standard, however I have significant reservations, as outlined above.
